# The association between metabolic syndrome and successful aging- using an extended definition of successful aging

**DOI:** 10.1371/journal.pone.0260550

**Published:** 2021-11-30

**Authors:** Yi-Hsuan Lin, Jeng-Min Chiou, Ta-Fu Chen, Liang-Chuan Lai, Jen-Hau Chen, Yen-Ching Chen

**Affiliations:** 1 Department of Family Medicine, Cheng Hsin General Hospital, Taipei, Taiwan; 2 Department of Family Medicine, School of Medicine, National Yang Ming Chiao Tung University, Taipei, Taiwan; 3 Institute of Statistical Science, Academia Sinica, Taipei, Taiwan; 4 Department of Neurology, National Taiwan University Hospital, Taipei, Taiwan; 5 Graduate Institute of Physiology, College of Medicine, National Taiwan University, Taipei, Taiwan; 6 Institute of Epidemiology and Preventive Medicine, College of Public Health, National Taiwan University, Taipei, Taiwan; 7 Department of Geriatrics and Gerontology, National Taiwan University Hospital, Taipei, Taiwan; 8 Department of Public Health, College of Public Health, National Taiwan University, Taipei, Taiwan; Università degli Studi di Milano, ITALY

## Abstract

**Objectives:**

To examine the association between metabolic syndrome (MetS) and successful aging among community-dwelling older adults.

**Methods:**

Adults aged ≥ 65 years who participated in the senior health checkup program at National Taiwan University Hospital during 2011–2013 were recruited (N = 467 at baseline). The participants were followed after 4 years and 6 years. MetS was assessed at baseline. Successful aging was evaluated at baseline, 4-year follow-up, and 6-year follow-up. We adopted an extended definition of successful aging, which was defined as three major domains: physiological, psychological, and sociological and economic domains. Generalized linear mixed models were used to assess the association between MetS and successful aging adjusting for time (follow-up years), age, sex, years of education, alcohol consumption and MetS×time interaction term.

**Results:**

The mean age of the study population was 72.9 (SD 5.5) years. The absence of baseline MetS had a positive effect on the probability of successful aging over six years. The absences of abdominal obesity, hyperglycemia, reduced high-density lipoprotein cholesterol, and hypertension were associated with the physiological successful aging. The absence of hypertension was the most significant predictor of physiological successful aging [aOR (95% CI) = 2.76 (1.67–4.58), *p*<0.001]. Significant increased trend was found in the overall and physiological successful aging across MetS status (No MetS, pre MetS, MetS; *P*_trend_ <0.001).

**Conclusions:**

We found that MetS is a risk factor of successful aging among community-dwelling older adults. Public health policy should aim at avoidance of MetS in order to facilitate successful aging in older population.

## Introduction

Aging is a global issue. According to the World Health Organization, the number of people ages 65 or older is expected to reach 1.5 billion in 2050 [1]. The number of children under age 5 was outnumbered by older adults aged 65+ globally in 2018 [2]. Taiwan has become an aged society (>14% of aged people) in 2018 [3]. The population aged 65+ in Taiwan has reached 3.9 million in 2021, and Taiwan is expected to be a super-aged society (>20% of aged people) by 2026 [3]. Aging is associated with chronic diseases, disability, and dependency, which are accompanied by increased health care cost and economic consequences [4]. Currently, health promotion for the aging population tends to focus on improvement or maintenance of function rather than on longevity. People can adapt well to aging when their health, vitality and social engagement can be maintained, and those variables are the key concepts of successful aging.

In 1997, Rowe and Kahn proposed the biomedical model of successful aging [5]. The model defines successful aging as the avoidance of disease and disability, preservation of cognitive and physical functions, and active engagement with life. The biomedical model is the most widely used definition for successful aging. However, achieving a disease-free status is unrealistic for most older adults. The biomedical model may not be applicable to most older people because multiple morbidities are common in older adults [6]. Psychosocial theory emphasizes social participation, life satisfaction, and positive interactions with others [7]. In a study of British people aged ≥ 50 years, the top three factors of successful aging were health, psychological well-being, and social roles and activities [7]. Another study of Taiwanese older adults found that the most important components of successful aging included physical health, being independent, family and social support, and economic security [8]. Therefore, successful aging is a multidimensional concept and a bio-psycho-social approach makes the definition of successful aging more complete.

Successful aging mainly includes physiological, psychological, sociological and environmental domains [9, 10]. Successful aging in the physiological domain indicates being free from diseases and functional impairments. The psychological domain includes cognitive function, depression, life satisfaction, self-acceptance, and emotional vitality [9]. The sociological domain is composed of interaction with one’s surroundings and participation in social activities and relationships with family. The environmental domain refers to external environmental factors, e.g., financial and economic security [10]. Studies researching successful aging have emerged in the past two decades. Although many factors had been related to or used to define successful aging [11], the association between metabolic syndrome (MetS) and successful aging remains unknown.

MetS, also known as “Syndrome X”, is a cluster of metabolic disorders including visceral obesity, insulin resistance, hypertension, and dyslipidemia [12]. The prevalence of MetS increases with age [13]. MetS increases the risk of type 2 diabetes mellitus, cardiovascular disease, and overall mortality [14]. MetS has been associated with limitation of mobility [15, 16], cognitive decline [17, 18], depressive symptoms and depression [19], and poor self-rated health [20]. Despite the wealth of existing literature, studies about the health consequences of MetS discussed successful aging fragmentally from individual components instead of making a comprehensive assessment of successful aging. There exists a research gap between MetS and successful aging.

To the best of our knowledge, this study is the first to investigate the association between MetS and successful aging in a community-based older population. Instead of traditional biomedical model, we adopted an extended definition of successful aging by using a multidimensional and comprehensive approach (including physiological, psychological, and sociological and economic domains). In addition to exploring the association of MetS with global and domain-specific successful aging, we further assessed whether some important variables (baseline age and inflammatory markers [C-reactive protein (CRP) and interleukin 6 (IL-6)] modified these associations). Through the identification of modifiable risk factors of successful aging, we hoped to facilitate health promotion in our rapidly aging society.

## Materials and methods

### Study design and population

This 6-year cohort study is part of an ongoing prospective cohort study “Taiwan Initiatives for Geriatric Epidemiological Research” (TIGER). The TIGER study was approved by the research ethics committee at National Taiwan University Hospital. We recruited the study population from community-dwelling older adults aged ≥ 65 years who participated in the senior health checkup program at National Taiwan University Hospital during 2011–2013 (baseline). A written informed consent was obtained before recruitment from the older adults who agreed to participate in the TIGER study. The participants were followed up after 4 years (2015–2017) and 6 years (2017–2019). A total of 467 participants with complete data of MetS and successful aging at baseline were included for analyses.

### Assessment of metabolic syndrome

MetS was evaluated at baseline according to the National Cholesterol Education Program’s Adult Treatment Panel III (ATP III) guidelines [21]. The criteria of MetS includes (1) abdominal obesity: waist circumference (for Asians) ≥ 90 cm in men and ≥ 80 cm in women; (2) hyperglycemia: serum fasting glucose level ≥ 100 mg/dl, or receiving drug treatment for elevated blood sugar; (3) reduced high-density lipoprotein cholesterol (HDL-C): serum HDL-C < 40 mg/dl in men and < 50 mg/dl in women or on cholesterol drug treatment; (4) elevated triglycerides: serum triglyceride ≥ 150 mg/dl or on drug treatment of hypertriglyceridemia; (5) hypertension: systolic blood pressure ≥ 130 mmHg, diastolic blood pressure ≥ 85 mmHg, or on antihypertensive drug treatment.

MetS was considered as present if three or more of the above diagnostic criteria were met. For MetS status, participants were divided into categories of no MetS, pre-MetS (one or two of diagnostic criteria) and MetS (≥ three diagnostic criteria) groups.

### Assessment of successful aging

**Fig 1:**
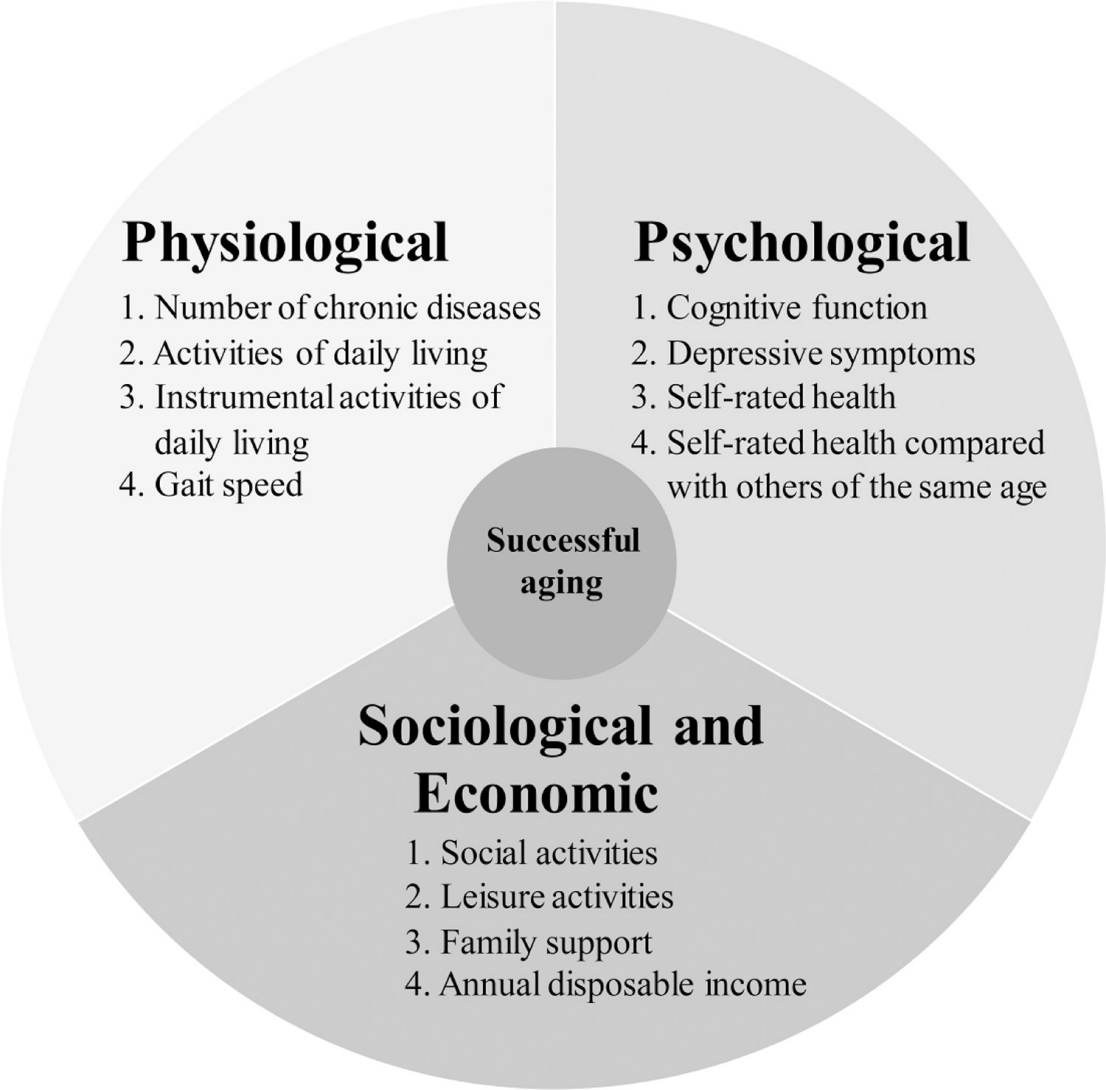
The definitions of successful aging.

Successful aging was evaluated at baseline, 4-year follow-up, and 6-year follow-up. The assessment of successful aging was based on literature review [7, 10]. We selected variables most commonly included in previous studies based on their biomedical role. In addition to the biomedical model, we also included the psycho-social domain of successful aging, which was less complete and not well defined previously. The extended definition of successful aging in this study included three major categories: physiological domain, psychological domain, and sociological and economic domain (Fig 1 and S1 Table). To make balanced information of three domains, we identified four corresponding variables for each domain.

The variables of the physiological domain included number of chronic diseases, activities of daily living (ADL) [22], instrumental activities of daily living (IADL) [23] and gait speed of Short Physical Performance Battery Protocol [24]. We considered eight chronic diseases that are common among older population, including hypertension, diabetes mellitus, stroke, heart disease, chronic obstructive pulmonary disease, osteoporosis, osteoarthritis, and Parkinson’s disease [9]. To test their gait speed, we asked participants to walk for 2.4 m at their normal pace [24]. The time (seconds) participants took to walk this distance was measured twice. The average gait speed was calculated for analyses.

The psychological domain was composed of cognitive function, depressive symptoms, self-rated health, and self-rated health compared with others of the same age. The cognitive function was assessed by the Montreal Cognitive Assessment-Taiwanese version (MoCA-T) [25]. MoCA-T is a more sensitive tool than the Mini-Mental State Examination for detecting mild cognitive impairment [26]. The 20-item measure of Center for Epidemiologic Studies Depression (CES-D) was used to identify participants with depressive symptoms. Depressive symptoms were deemed present if participants reported a history of depression, usage of anti-depressive agents, or had a CES-D score ≥ 16 [27]. The self-rated health and self-rated health compared with others of the same age were both self-reported, with scales ranging from 1 (best) to 5 (worst).

The variables in the sociological and economic domain were frequency of participation in social activities and leisure activities, family support, and annual disposable income. Frequency of participation in social and leisure activities was measured on an ordinal scale ranging from 0 (never) to 6 (everyday). Family support was assessed by living status (lived alone or in a nursing home *vs*. lived with family members) and frequency of visits from family and friends. Participants were categorized as having good family support if they did not live alone, did not live in a nursing home, or if the frequency of visits from family and friends was greater than once per week. Economic security was evaluated via self-reported annual disposable income, which ranged from 1 [< $10,000 United States dollar (USD)] to 4 (greater than $33,333 USD, which indicates financially security in Taiwan).

Because the scales and effects are different across variables used for defining successful aging, we standardized all variables for defining successful aging and made them in the same direction. These standardized scores were summed up to a composite score for each domain of successful aging. A higher successful aging score represented a better condition of successful aging. The cutoff point of the successful aging score was based on the tertiles of the study participants in this study (n = 467). Participants in the highest tertile (T3: successful aging score ≥ 2.33) were defined as the successful aging group (n = 156), and the remaining two tertiles were classified as the usual aging group. The cutoff value of the successful aging score at baseline was applied to the 4-year follow-up and 6-year follow-up.

### Other variables

The measures of blood pressure (mmHg), body height (cm), body weight (kg), and waist circumference (cm) were obtained by well-trained nurses. Self-reported information included marital status, years of education, medical history, medication use, diet intake, physical activity, smoking status, and alcohol consumption. Smoking status and alcohol consumption were defined by ever or current use. Dietary intake was recorded by a 44-item food frequency questionnaire. We converted the information of diet intake to the modified Alternative Healthy Eating Index (mAHEI) to evaluate diet quality [28, 29]. The International Physical Activity Questionnaire (IPAQ) was used to quantify physical activity as the metabolic equivalent of task (MET) in the past week [30].

### Laboratory assay

Serum HDL-C (mg/dl), triglycerides (mg/dl), and fasting glucose (mg/dl) were analyzed by automatic analyzer. Serum CRP (mg/dl) was analyzed by TBA-200FR (Toshiba Medical System, Japan). Serum IL-6 (pg/ml) was tested by Tecan Infinite M200 multimode reader (Tecan, Männedorf, Switzerland). Participants with the highest tertile (T3) of CRP and IL-6 level at baseline were categorized as high-level CRP and high-level IL-6 groups.

### Statistical analysis

For the descriptive analyses, chi-square tests and Fisher’s exact tests were used for categorical variables and Mann-Whitney U tests were used for continuous variables to compare the distribution of variables by the presence of MetS. The generalized linear mixed model (GLMM) [31] with random intercept was used to estimate adjusted odds ratios (aOR) and 95% confidence intervals (95% CI) for the association between MetS and successful aging, adjusting for time (follow-up years), age, sex, years of education, alcohol consumption and MetS×time interaction. A two-sided *p* < 0.05 was considered statistically significant. We further performed stratified analyses by baseline age, serum CRP levels, and IL-6 levels. A *P*_interactions_ < 0.1 was regarded as the presence of an interaction. Statistical analyses were performed with SAS version 9.4 (SAS Institute Inc., Cary, NC, USA).

### Sensitivity analysis

To compare findings using our extended definition of successful aging with those using traditional biomedical model [5], we used the following criteria (1) free from chronic diseases including hypertension, diabetes mellitus, stroke, heart disease, chronic obstructive pulmonary disease, osteoporosis, osteoarthritis, and Parkinson’s disease, (2) no impairment of ADL, (3) no cognitive impairment, which was represented by the MoCA-T ≥ 24 [25], (4) no depressive symptoms, which was represented by no history of depression, no usage of anti-depressive agents, and the CES-D score < 16. Participants who were eligible for the criteria were deemed as successful aging group in the sensitivity analysis.

## Results

The mean age of the study population was 72.9 (SD 5.5) years, and 54.8% of participants were women. The median follow-up period was 5.6 years. At baseline, 39.2% (n = 183) of the study population had MetS (Table 1). Compared with older adults with MetS, those without MetS were less likely to be women, less likely to have depressive symptoms, and more likely to be educated. They also had a better diet quality, a lower body mass index, a lower level of mean serum CRP and IL-6, and fewer chronic diseases, e.g., hypertension, diabetes mellitus, hyperlipidemia and cardiac disease.

**Table 1:** Characteristics of the study population at baseline (2011–2013).

	No MetS	MetS	*P*
	n = 284 (60.8%)	n = 183 (39.2%)	
Age years, mean (SD)	72.9 (5.5)	73.0 (5.5)	0.92
Women, n (%)	143 (50.1)	113 (61.8)	0.02
Married, n (%)	231 (81.3)	144 (78.7)	0.48
Cigarette smoking^a^ (y/n), n (%)	36 (12.7)	34 (18.6)	0.08
Alcohol consumption^a^ (y/n), n (%)	57 (20.1)	36 (19.7)	0.92
Physical activity (MET), mean (SD)	1749.6 (1406.9)	1561.2 (1425.2)	0.06
Years of education, mean (SD)	13.9 (3.6)	13.0 (4.0)	0.01
MoCA-T^b^, mean (SD)	26.5 (3.0)	25.8 (3.7)	0.15
Diet quality-mAHEI score^c^, mean (SD)	37.6 (7.6)	34.8 (8.0)	0.001
Daily calories (kcal), mean (SD)	1600.8 (370.0)	1522.3 (391.6)	0.05
BMI (kg/m2), mean (SD)	23.0 (2.5)	25.5 (3.0)	<0.001
Follow-up years, mean (SD)	4.7 (2.1)	4.0 (2.5)	0.01
Depressive symptoms^d^ (y/n), n (%)	23 (8.1)	26 (14.2)	0.04
Annual disposable income > $33,333 USD^e^, n (%)	135 (47.5)	81 (44.3)	0.49
CRP (mg/dl), mean (SD)	0.18 (0.69)	0.22 (0.28)	<0.001
IL-6 (pg/ml), mean (SD)	2.30 (2.19)	2.63 (2.22)	0.03
History of chronic disease			
Hypertension, n (%)	112 (39.4)	127 (69.4)	<0.001
Diabetes mellitus, n (%)	13 (4.6)	52 (28.4)	<0.001
Hyperlipidemia, n (%)	103 (36.3)	122 (66.7)	<0.001
Cardiac disease, n (%)	64 (22.5)	60 (32.8)	0.01
Stroke, n (%)	5 (1.8)	4 (2.2)	0.74
Parkinson’s disease	3 (1.1)	1 (0.6)	1.00
COPD, n (%)	26 (9.2)	22 (12.0)	0.32
Arthritis, n (%)	86 (30.3)	63 (34.4)	0.35
Osteoporosis, n (%)	97 (34.2)	54 (29.5)	0.29

Abbreviations: MetS, metabolic syndrome; SD, standard deviation; y/n, yes or no; MET, metabolic equivalent of task; MoCA-T, Montreal Cognitive Assessment-Taiwanese version; mAHEI, modified Alternative Healthy Eating Index; BMI, body mass index; USD, United States dollar; CRP, C-reactive protein; IL-6, interleukin 6; COPD, chronic obstructive pulmonary disease; CES-D, Center for Epidemiologic Studies Depression.

^a^ Smoking and alcohol consumption indicated ever or current users.

^b^ Participants with MoCA-T score ≥ 24 were categorized as normal cognition.

^c^ mAHEI score (ranging from 0–70) includes seven food components (i.e., fruits, vegetables, soy protein, fish/(meat + eggs), whole grain, fried foods, and alcohol). Nuts were removed from the component “nuts and soy protein” because nuts are not constantly consumed by older Taiwanese adults [29].

^d^ Depressive symptoms (yes/no) were defined as at least one of the following three factors: self-reported diagnosis, use of anti-depressive agents, or CES-D score ≥ 16.

^e^ Annual disposable income greater than $33,333 USD indicates financially security in Taiwan.

Chi-square tests and Fisher’s exact tests were used for categorical variables.

Mann-Whitney U tests were used for continuous variables.

Compared with the MetS group, older adults without MetS at baseline had a higher probability of successful aging [aOR (95% CI) = 2.71 (1.67–4.39); Table 2]. Older adults without MetS performed better on the physiological and psychological successful aging [aOR (95% CI) = 5.03 (3.04–8.34) and 1.67 (1.06–2.65), respectively]. The absences of abdominal obesity and hyperglycemia, as well as reduced HDL-C and hypertension were associated with physiological successful aging [aOR (95% CI) = 1.65 (1.02–2.67), 1.68 (1.03–2.76), 2.21 (1.34–3.63), and 2.76 (1.67–4.58), respectively]. Among components of MetS, the absence of hypertension was the most significant predictor of physiological successful aging (*p* < 0.001).

**Table 2:** The associations between MetS and successful aging over six years (n = 467).

Variables	Overall successful aging ^a^		Domains of successful aging					
			Physiological		Psychological		Sociological and economic	
	Adjusted OR (95% CI)	*P*	Adjusted OR (95% CI)	*P*	Adjusted OR (95% CI)	*P*	Adjusted OR (95% CI)	*P*
**Model 1:**								
No MetS	2.71 (1.67–4.39)	<0.001	5.03 (3.04–8.34)	<0.001	1.67 (1.06–2.65)	0.03	0.95 (0.62–1.44)	0.80
No MetS×time	1.00 (0.90–1.12)	0.95	0.96 (0.84–1.10)	0.55	0.94 (0.84–1.05)	0.29	1.09 (0.98–1.21)	0.13
**Model 2:**								
No abdominal obesity	1.31 (0.80–2.13)	0.28	1.65 (1.02–2.67)	0.04	1.15 (0.71–1.84)	0.57	1.63 (1.04–2.56)	0.03
No hyperglycemia	1.25 (0.76–2.06)	0.38	1.68 (1.03–2.76)	0.04	1.08 (0.66–1.75)	0.77	1.17 (0.75–1.82)	0.50
No reduced HDL-C	1.41 (0.86–2.32)	0.17	2.21 (1.34–3.63)	0.002	0.96 (0.56–1.55)	0.85	0.68 (0.43–1.06)	0.09
No elevated triglycerides	1.56 (0.82–2.98)	0.17	1.30 (0.66–2.56)	0.44	1.95 (1.02–3.74)	0.04	0.76 (0.44–1.30)	0.31
No hypertension	1.81 (1.06–3.08)	0.03	2.76 (1.67–4.58)	<0.001	1.66 (0.98–2.79)	0.06	1.10 (0.68–1.78)	0.69
No abdominal obesity×time	1.01 (0.90–1.14)	0.82	0.95 (0.85–1.07)	0.38	1.00 (0.89–1.11)	0.98	0.91 (0.82–1.01)	0.08
No hyperglycemia×time	1.04 (0.94–1.16)	0.46	0.96 (0.85–1.07)	0.47	1.01 (0.90–1.13)	0.87	1.06 (0.94–1.19)	0.34
No reduced HDL-C×time	1.01 (0.90–1.14)	0.87	1.02 (0.89–1.17)	0.74	1.04 (0.93–1.17)	0.47	1.04 (0.93–1.16)	0.54
No elevated triglycerides×time	0.90 (0.77–1.05)	0.18	1.05 (0.86–1.27)	0.64	0.84 (0.71–0.98)	0.03	1.07 (0.92–1.26)	0.38
No hypertension×time	0.98 (0.88–1.10)	0.76	0.98 (0.87–1.11)	0.79	0.94 (0.84–1.04)	0.21	0.99 (0.89–1.11)	0.90

Abbreviations: OR, odds ratio; CI, confidence interval; MetS, metabolic syndrome; HDL-C, high-density lipoprotein cholesterol; T, tertile; GLMM, generalized linear mixed model.

^a^ The cutoff point of the successful aging score (range: -24.76 to 9.12) was based on the tertiles of the study participants. Participants in the highest tertile were defined as the successful aging group (T3: score ≥ 2.33).

The analyses were conducted by GLMM.

Model 1 and Model 2 were adjusted for time, age, sex, years of education, alcohol consumption and MetS×time interaction.

Older adults without abdominal obesity were more likely to age successfully in the sociological and economic domain [aOR (95% CI) = 1.63 (1.04–2.56)]. The absence of elevated triglycerides was associated with psychological successful aging [aOR (95% CI) = 1.95 (1.02–3.74)], but the protective effect reduced over time [no elevated triglycerides×time interaction: aOR (95% CI) = 0.84 (0.71–0.98)].

**Fig 2:**
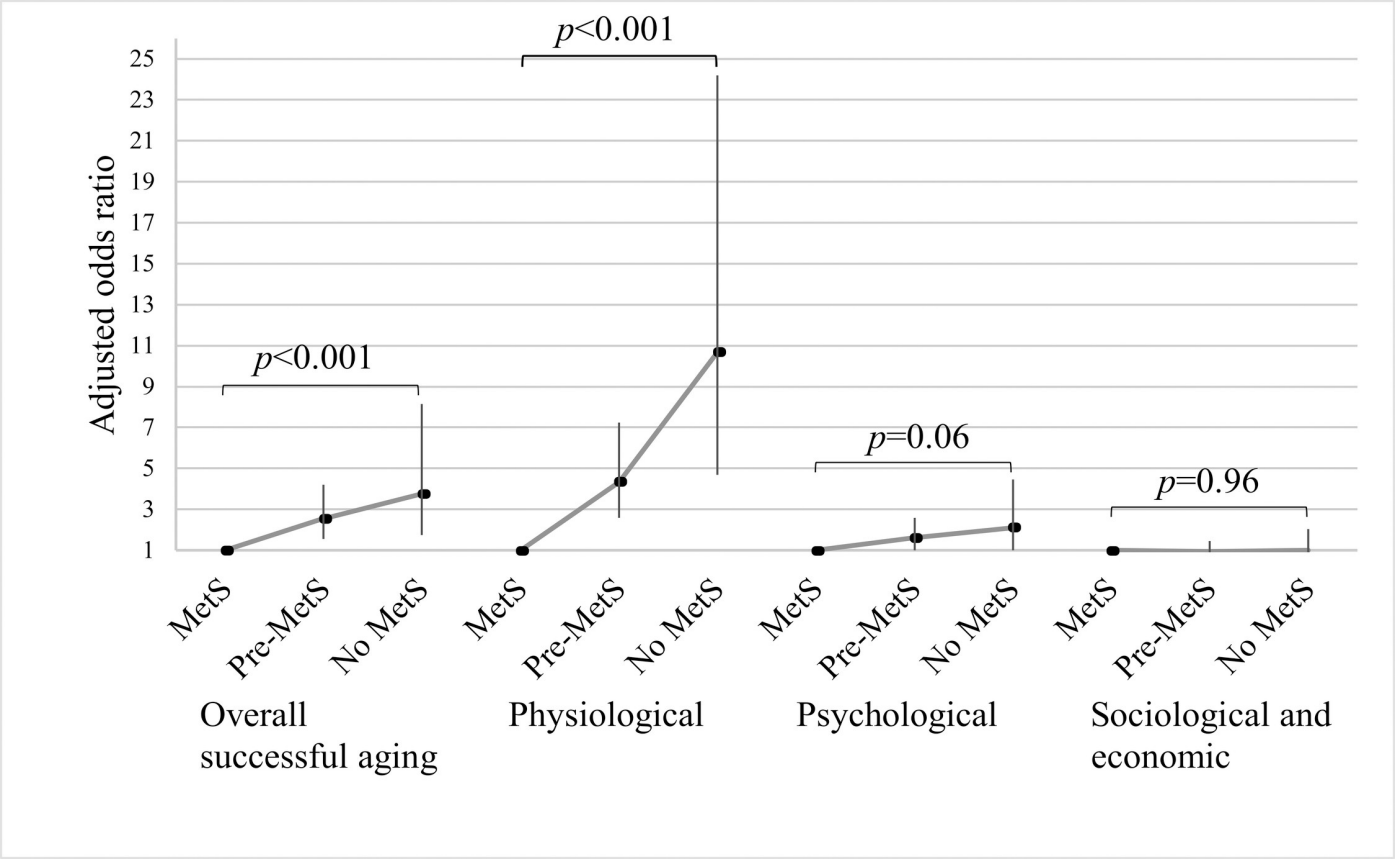
Adjusted odds ratio and 95% confidence interval for the association between MetS status and successful aging. Abbreviations: MetS, metabolic syndrome; Ref., reference; GLMM, generalized linear mixed model. GLMM model: Successful aging~ MetS status+ Age+ Sex+ Years of education+ Alcohol+ Time+ MetS status×time. The *p* values compared the differences across MetS status [no MetS, pre-MetS, and MetS (ref.)].

Fig 2 shows the association of MetS status with both overall and domain-specific successful aging. Significant positive trends in successful aging were found when MetS severity decreased from MetS, pre-MetS to no MetS for overall [pre-MetS *vs*. MetS: aOR (95% CI) = 2.54 (1.54–4.20); no MetS *vs*. MetS: 3.76 (1.74–8.14), *P*_trend_ < 0.001] and physiological successful aging [pre-MetS *vs*. MetS: aOR (95% CI) = 4.33 (2.58–7.25); no MetS *vs*. MetS: 10.67 (4.70–24.20), *P*_trend_ < 0.001].

Table 3 demonstrates the association of HDL-C and hypertension with physiological successful aging stratified by baseline age groups (65–74 years *vs*. 75 years and older), CRP groups [low- *vs*. high-level (≥ 0.14 mg/dl)] and IL-6 groups [low- *vs*. high-level (≥ 2.2 pg/ml)]. Among participants with high-level IL-6, the absence of low HDL-C was associated with physiological successful aging [aOR (95% CI) = 4.41 (1.75–11.15), *P*_interactions_ = 0.05]. Among participants older than 75 years old, the absence of hypertension was associated with physiological successful aging [aOR (95% CI) = 4.12 (1.20–14.22), *P*_interactions_ < 0.001]. Among participants aged 65–74 years, the absence of hypertension was associated with physiological successful aging [aOR (95% CI) = 2.55 (1.44–4.52)].

**Table 3:** The associations of hypertension and HDL-C with physiological successful aging over six years stratified by important variables (baseline age, CRP, and IL-6).

Status of MetS components	Physiological successful aging ^a^ /Usual aging	Adjusted OR (95% CI)	Physiological successful aging ^a^ /Usual aging	Adjusted OR (95% CI)	*P*_interactions_
		**Age 65–74**		**Age ≥ 75**	
Hypertension	62/139	Ref.	24/122	Ref.	<0.001
No hypertension	55/46	2.55 (1.44–4.52)	9/10	4.12 (1.20–14.22)	
		**Low-level CRP**		**High-level CRP** ^b^	
Hypertension	60/165	Ref.	26/96	Ref.	0.36
No hypertension	48/41	2.51 (1.39–4.51)	16/15	3.98 (1.32–11.99)	
		**Low-level IL-6**		**High-level IL-6** ^b^	
Hypertension	54/171	Ref.	32/90	Ref.	0.25
No hypertension	48/37	3.89 (2.13–7.13)	16/19	1.39 (0.52–3.68)	
		**Age 65–74**		**Age ≥ 75**	
Reduced HDL-C	32/95	Ref.	8/70	Ref.	0.89
No reduced HDL-C	85/95	1.92 (1.05–3.52)	25/62	3.49 (1.19–10.19)	
		**Low-level CRP**		**High-level CRP** ^b^	
Reduced HDL-C	24/100	Ref.	16/65	Ref.	0.60
No reduced HDL-C	84/106	2.16 (1.17–3.99)	26/46	2.58 (1.02–6.55)	
		**Low-level IL-6**		**High-level IL-6** ^b^	
Reduced HDL-C	30/99	Ref.	10/66	Ref.	0.05
No reduced HDL-C	72/109	1.65 (0.89–3.06)	38/43	4.41 (1.75–11.15)	

Abbreviations: HDL-C, high-density lipoprotein cholesterol; CRP, C-reactive protein; IL-6, interleukin 6; OR, odds ratio; CI, confidence interval; T, tertile; GLMM, generalized linear mixed model.

^a^ The cutoff point of the physiological successful aging score (range: -15.92 to 3.50) was based on the tertiles of the study participants. Participants in the highest tertile were defined as the physiological successful aging group (T3: score ≥ 1.26).

^b^High-level CRP (≥ 0.14 mg/dl) and high-level IL-6 (≥ 2.2 pg/ml) indicated the highest tertile (T3) of CRP and IL-6 level at baseline; Low-level CRP and low-level IL-6 indicated the lower tertiles (T1+T2) of CRP and IL-6 level at baseline.

GLMM models were adjusted for MetS components, time (follow-up years), age, sex, years of education, alcohol consumption and interaction term with time for each MetS components.

*P*_interactions_< 0.1 was considered as statistically significant.

The results of sensitivity analysis were similar to the main analyses. Older adults without MetS were more likely to aged successfully than MetS group [aOR (95% CI) = 6.38 (2.02–20.18); Table 4].

**Table 4:** Sensitivity analysis for the associations between MetS and successful aging over six years (n = 467) using the biomedical model of successful aging.

	Adjusted OR (95% CI)	*P*
No MetS	6.38 (2.02–20.18)	0.002
No MetS×time	0.93 (0.66–1.31)	0.67
Age (years)	0.93 (0.87–0.99)	0.02
Women	0.78 (0.40–1.53)	0.47
Education (years)	1.03 (0.93–1.13)	0.57
Alcohol consumption	0.56 (0.22–1.42)	0.22
Time (Follow-up years)	0.95 (0.80–1.13)	0.57

Abbreviations: OR, odds ratio; CI, confidence interval; MetS, metabolic syndrome; ADL, activity of daily living; GLMM, generalized linear mixed model.

In the biomedical model, successful aging was defined as the absence of chronic diseases, disability of ADL, cognitive impairment, and depressive symptoms.

The analyses were conducted by GLMM.

## Discussions

This study found MetS is a risk factor of successful aging among community-dwelling older adults. We also found a significant increasing trend in successful aging as MetS severity decreased. This study is the first to investigate the association between MetS and successful aging.

Because of the multidimensional construct of successful aging, we adopted an extended definition of successful aging including physiological, psychological, sociological and economic domains. We further used the traditional biomedical model of successful aging for sensitivity analysis. Compare with our extended definition on successful aging, the detrimental effect of MetS was stronger when the traditional biomedical model was adopted. However, our extended definition is more applicable because most older adults tend to have at least one diseases (prevalence = 64.5% in Taiwanese older adults aged 65+ at 2010) [32].

The prevalence of MetS ranged from 32.9–41.4% in Taiwanese older adults aged 70–79 years old [13], which is consistent with the prevalence observed in this study (39.2%). MetS is known to increase the risk of physical disability [15, 16]. We found strong associations of MetS and MetS components with physiological successful aging. The results were consistent with a previous study [16], of which central obesity, elevated blood glucose, low HDL-C, and hypertension were associated with mobility or IADL limitations. A 4.5-year cohort study in United States also found all MetS components to be associated with mobility limitations [33]. Similarly, a three-year cohort study in Italy found abdominal obesity, low HDL-C, and high blood pressure to be associated with prevalent disability in ADL [34].

Among the components of MetS, hypertension had the strongest negative effect on physiological successful aging. Age significantly modified the association between hypertension and physiological successful aging. Aging is a continuous process of deteriorating physical function, which is characterized by chronic inflammation [35]. The ability of recovery from inflammation is impaired in older adults [36]. Therefore, the accumulated aging-related oxidative stress and increased inflammation results in chronic diseases, e.g., hypertension, cardiovascular disease, diabetes mellitus, cognitive decline and dementia, etc [35]. Accordingly, the prevalence of hypertension increased with age [37] and the benefits from absence of hypertension also increased with age.

In addition to the physiological domain, we found MetS was also associated with psychological successful aging. We found absence of elevated triglycerides increased the probability of psychological successful aging, which was consistent with a previous study [38]. However, this association decreased over time, which may imply the important role of time in the aging process. Previous cross-sectional and cohort studies have shown MetS and its components to be associated with increased risk of dementia and cognitive impairment [17, 18, 39]. Furthermore, MetS and its components increased the risk of depression and depressive symptoms [38, 40, 41], poor self-rated health [20, 42] (components of psychological successful aging), and low social support (a component of sociological successful aging) [43].

Our study created a variable, "MetS status" (no MetS, pre-MetS, and MetS) to show the "severity" of MetS, which is consistent with previous research [15, 33] that found the risk of disability increased as the number of MetS components increased. The pathogenesis of MetS is complex and the effects of MetS components are usually correlated [33]. Therefore, some studies have assessed and reported synergistic interactions between MetS components and other health outcomes, such as insulin resistance [44], cardiovascular disease [45], and diabetes mellitus [46]. Taken together, the cluster of MetS components had a synergistic harmful effect on not only vascular outcomes but also successful aging.

MetS is a constellation of metabolic abnormalities, characterized by central obesity and insulin resistance [47]. The metabolic disorders are associated with low-level chronic inflammation and oxidative stress [48]. Metabolic disorders and inflammatory processes lead to the generation of reactive oxygen species and subsequent degradative processes, which increase disease risk and accelerate the aging process and functional decline [49, 50]. Prior studies have reported that high-level inflammatory markers, e.g., CRP and IL-6, to be associated with poor physical performance in older adults [51, 52]. Furthermore, low HDL-C enhanced the association between inflammation and physical function in older adults [52]. Our study also found the level of IL-6 modified the effect of low HDL-C on physiological successful aging, which suggests inflammation plays a role in the relationship between MetS and successful aging.

This study had several strengths. First, this study is the first to explore the relationship between MetS and successful aging. The definition of successful aging in this study was comprehensive and included multidimensional factors. Second, the study participants were relatively healthy older adults so our results are generalizable to primary prevention of the general population. In addition, we used GLMM to control the autocorrelation of repeated measures data and adjusted for important covariates. We also tried to adjust for some variables that may affect successful aging in the models, e.g., physical activity, marital status, smoking status, diet pattern (mAHEI) and daily calories. However, these covariates did not reach statistical significance. Because the importance of establishing a parsimonious model, these variables were not adjusted in the final models. Finally, our study was a six-year longitudinal design, which provided a greater amount of evidence than cross-sectional studies for the relationship between MetS and successful aging.

This study had some limitations. First, the study participants were recruited from older adults attending a senior health checkup program in a hospital, which may have resulted in participation bias. However, this bias diminished as participants followed up over time [53]. Second, our participants had higher socioeconomic status and health literacy compared with the average older population in Taiwan. Lower socioeconomic status tends to predict poorer health status [54], so findings from this population are more likely to underestimate the effect. Third, reverse causation was possible because the definitions of MetS and successful aging partly overlapped. The number of chronic diseases was a variable of physiological successful aging and the included diseases (hypertension and diabetes mellitus) are components of MetS. Spearman’s rank correlation coefficient was calculated between the number of MetS components and the number of chronic diseases and yielded a low positive correlation (0.37). Fourth, data for MetS in the follow-ups were lacking. MetS was only assessed at baseline. Therefore, we used baseline MetS to predict successful aging over six years.

In conclusion, this study found MetS to be a risk factor of successful aging among community-dwelling older adults. The trend of increased successful aging correlated with decreased MetS severity. The prevention of MetS could serve as a public health priority to facilitate successful aging in older populations.

## Supporting information

S1 Table Variables and scoring strategy of successful aging. (DOCX)
