# Supplementary material for: The association between metabolic syndrome and successful aging- using an extended definition of successful aging
Source: PLoS One. 2021 Nov 30;16(11):e0260550. doi: 10.1371/journal.pone.0260550 (PMC8631634; doi:10.1371/journal.pone.0260550)
Supplement: S1 Table — (DOCX) [file pone.0260550.s001.docx]

**S1 Table. Variables and scoring strategy of successful aging.**

| Domains of  successful aging | Variables | Scoring criteria | Types of  variables | Range |
| --- | --- | --- | --- | --- |
| Physiological | Number of chronic diseases | Hypertension, diabetes mellitus, stroke, heart disease, chronic obstructive pulmonary disease, osteoporosis, osteoarthritis and Parkinsonism | Continuous | 0-8 |
|  | Activities of daily living | Eating, transferring from bed to chair, personal hygiene, using the toilet, bathing, walking on surface level, taking stairs, dressing, continence | Continuous | 0-100 |
|  | Instrumental activities of daily living | Shopping, doing housework, handling finances, preparing food, handling their own transportation, using telephone, doing laundry, and managing medications | Continuous | 0-8 |
|  | Gait speed | The average gait speed (seconds) of walking twice for 2.4 m at normal pace | Continuous | - |
| Psychological | Cognitive function (MoCA-T) | Visuospatial/executive, naming, memory, attention, language, abstraction, delayed recall, orientation | Continuous | 0-30 |
|  | Depressive symptoms | 1: At least one of the following three factors: self-reported diagnosis, use of anti-depressive agents, or CES-D score≥ 16;  0: None of above factors | Nominal | 0, 1 |
|  | Self-rated health | 1. Excellent; 2. Good; 3. Fair; 4. Poor; 5. Very poor | Ordinal | 1-5 |
|  | Self-rated health compared with people of the same age | 1. Excellent; 2. Good; 3. Fair; 4. Poor; 5. Very poor | Ordinal | 1-5 |
|  |  |  |  |  |
| Sociological and  economic | Frequency of social activity | 0. Never; 1. Several times per year; 2. Less than once per month; 3. Two or three times per month; 4. Once per week; 5. Two or three times per week; 6. Everyday or almost everyday | Ordinal | 0-6 |
|  | Frequency of leisure activity | 0. Never; 1. Several times per year; 2. Less than once per month; 3. Two or three times per month; 4. Once per week; 5. Two or three times per week; 6. Everyday or almost everyday | Ordinal | 0-6 |
|  | Family support | 1: Lived with spouse, children, grand-children or relatives; lived alone or lived in a nursing home with the frequency of visits from family and friends ≥ once per week  0: Lived alone or lived in a nursing home with the frequency of visits from family and friends <once per week | Nominal | 0, 1 |
|  | Annual disposable income | 1. < $10,000 USD; 2. $10,000~26,666 USD; 3. $26,666~33,333 USD; 4. > $33,333 USD | Ordinal | 1-4 |

Abbreviations: MoCA-T, Montreal Cognitive Assessment-Taiwanese version; CES-D, Center for Epidemiologic Studies Depression; USD, United States dollar.
